# Mobile Phone Radiation Induces Reactive Oxygen Species Production and DNA Damage in Human Spermatozoa *In Vitro*

**DOI:** 10.1371/journal.pone.0006446

**Published:** 2009-07-31

**Authors:** Geoffry N. De Iuliis, Rhiannon J. Newey, Bruce V. King, R. John Aitken

**Affiliations:** 1 ARC Centre of Excellence in Biotechnology and Development, Callaghan, New South Wales, Australia; 2 School of Environmental and Life Sciences, The University of Newcastle, Callaghan, New South Wales, Australia; 3 School of Mathematical and Physical Sciences, The University of Newcastle, Callaghan, New South Wales, Australia; East Carolina University, United States of America

## Abstract

**Background:**

In recent times there has been some controversy over the impact of electromagnetic radiation on human health. The significance of mobile phone radiation on male reproduction is a key element of this debate since several studies have suggested a relationship between mobile phone use and semen quality. The potential mechanisms involved have not been established, however, human spermatozoa are known to be particularly vulnerable to oxidative stress by virtue of the abundant availability of substrates for free radical attack and the lack of cytoplasmic space to accommodate antioxidant enzymes. Moreover, the induction of oxidative stress in these cells not only perturbs their capacity for fertilization but also contributes to sperm DNA damage. The latter has, in turn, been linked with poor fertility, an increased incidence of miscarriage and morbidity in the offspring, including childhood cancer. In light of these associations, we have analyzed the influence of RF-EMR on the cell biology of human spermatozoa in vitro.

**Principal Findings:**

Purified human spermatozoa were exposed to radio-frequency electromagnetic radiation (RF-EMR) tuned to 1.8 GHz and covering a range of specific absorption rates (SAR) from 0.4 W/kg to 27.5 W/kg. In step with increasing SAR, motility and vitality were significantly reduced after RF-EMR exposure, while the mitochondrial generation of reactive oxygen species and DNA fragmentation were significantly elevated (*P*<0.001). Furthermore, we also observed highly significant relationships between SAR, the oxidative DNA damage bio-marker, 8-OH-dG, and DNA fragmentation after RF-EMR exposure.

**Conclusions:**

RF-EMR in both the power density and frequency range of mobile phones enhances mitochondrial reactive oxygen species generation by human spermatozoa, decreasing the motility and vitality of these cells while stimulating DNA base adduct formation and, ultimately DNA fragmentation. These findings have clear implications for the safety of extensive mobile phone use by males of reproductive age, potentially affecting both their fertility and the health and wellbeing of their offspring.

## Introduction

Male infertility is a distressingly common condition affecting about 1 in 20 of the male population [1]. In a majority of cases, the male partner produces sufficient numbers of spermatozoa to achieve fertilization but there are functional defects in these cells that prevent conception from occurring [2]. Despite several decades of research, the causes of such functional deficiencies in human spermatozoa remain largely unresolved. However, one contributory factor that has recently emerged is the quality of the sperm DNA delivered to the oocyte at the moment of fertilization [3]. Fragmentation of DNA in the male germ line has been associated with impaired fertilization, poor embryonic development, high rates of miscarriage and an increased incidence of morbidity in the offspring, including childhood cancer [3], [4]. In view of the seriousness of these clinical outcomes, attention has recently focused on the environmental and genetic factors that might be involved in the aetiology of DNA damage in the male germ line.

These investigations have suggested that one of the environmental factors potentially involved in the etiology of DNA damage in human spermatozoa is an increased exposure to radio-frequency electromagnetic radiation (RF-EMR) emitted from mobile phones. This association was initially suggested by an epidemiological study which found negative correlations between mobile phone usage and various attributes of semen quality, particularly motility [5]. This was immediately followed by an experimental study involving exposure of male mice to RF-EMR, which revealed a significant impact on the integrity of both the mitochondrial and nuclear genomes [6]. Recently, the negative impact of mobile phone usage on semen quality in human males was confirmed in a study that found the duration of exposure to be correlated with defects in sperm count, motility, viability, and normal morphology [7]. In light of these data, there is now an urgent need to determine whether exposure of human spermatozoa to RF-EMR can also induce DNA damage and to resolve the cellular mechanisms involved.

Several studies have found an association between human health and exposure to RF-EMR, with emphasis on a range of clinical conditions including childhood leukaemia, brain tumours, genotoxicity and neurodegenerative disease [8], [9]. While the cellular mechanisms underpinning these effects have not been completely resolved, it has been suggested that oxidative stress could be a key factor [10]. However, extensive analysis of the importance of oxidative stress in mediating the pathological effects of RF-EMR has generated conflicting results, possibly due to differences in the fundamental redox susceptibility of the cell lines employed in these analyses [11]. In this context, it is significant that human spermatozoa are uniquely sensitive to oxidative stress for a variety of reasons. Firstly, these cells are largely devoid of the cytoplasm that in somatic cells houses the antioxidant enzymes that offer a first line of defense against free radical attack [12]. Secondly, these cells possess abundant targets for the induction of peroxidative damage including polyunsaturated fatty acids and DNA [12]–[14]. Thirdly, these cells are professional generators of reactive oxygen species, that appear to emanate largely from the sperm mitochondria and, possibly, plasma membrane NAD(P)H oxidases [15], [16]. Thus if any cell type would be vulnerable to the oxidative stress reportedly generated on exposure to RF-EMR, it would be human spermatozoa.

In light of these considerations, we have conducted a careful analysis of the biological consequences of exposing human spermatozoa to RF-EMR. The study design involved overnight exposure to RF-EMR at a defined frequency (1.8 GHz), over a range of SAR values that both covered the emission characteristics of mobile phones and generated sufficient dose-response data to shed light on the underlying pathophysiological mechanisms. Moreover, the temperature of the incubations was maintained at 21°C to avoid any secondary heating effects. The results clearly demonstrate that exposure to this type of radiation not only stimulates free radical generation by the sperm mitochondria but also creates a state of oxidative stress characterized by the formation of oxidative base adducts and DNA fragmentation. These data clearly have important implications for the safety of mobile phone use and highlight the potential importance of RF-EMR in the etiology of male infertility and childhood disease.

## Results

### RF-EMR disrupts human sperm motility and vitality and induces intracellular reactive oxygen species (ROS) production

**Figure 1:**
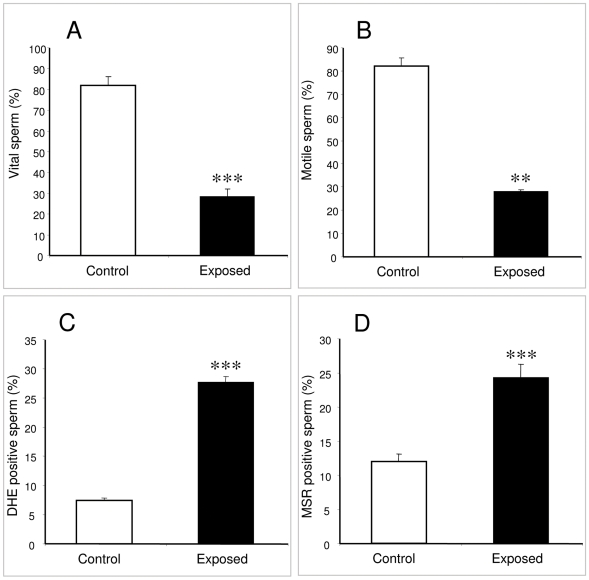
RF-EMR exposure decreases motility and vitality of human sperm while also inducing intracellular ROS. Percoll-purified spermatozoa (5×10^6^ cells) were suspended in 1 ml BWW in a 35 mm Petri dish and placed within the waveguide while control cells placed outside the waveguide. A frequency of 1.8 GHz at a SAR of 27.5 W/kg was used and all samples were incubated for 16 h at 21°C. A, Sperm vitality was significantly reduced from the control value of 82%±4% to 29%±4% for the exposed cells (***p<0.001). B, Sperm motility was also significantly reduced from the control value of 82%±4% to 28%±1% (**p<0.01). C, ROS production was increased after RF-EMR exposure such that 28%±1% of the cells were producing ROS, while only 7%±0.4% of the controls contributed to ROS production (***p<0.001). D, 24%±1% of the exposed cells generated mitochondrial ROS, while the only 12%±1% of the control cells produced ROS from this source (***p<0.001). All results are based on 4 independent samples.

In an initial experiment, functional human spermatozoa isolated from the high density region of Percoll gradients and suspended in BWW medium were exposed to RF-EMR at an SAR of 27.5 W/kg. This exposure induced a highly significant decline in both vitality (p<0.001; Figure 1A) and motility (p<0.01; Figure 1B) compared with the unexposed controls. Exposed spermatozoa also produced significantly higher amounts of ROS than background levels as measured by both the dihydroethidium (DHE) (p<0.001; Figure 1C) and MitoSOX red (MSR) probes (p<0.001; Figure 1D) suggesting that free radical generation had been initiated as a consequence of RF-EMR and that the mitochondria were significantly involved in this response.

### RF-EMR has a negative impact on human spermatozoa over a range of SAR values

In light of these results we then extended the range of SAR values over which the consequences of RF-EMR radiation were examined (0.4 W/kg–27.5 W/kg) to include the values covered by conventional mobile phones (0.5 W/kg–1.5 W/kg).

**Figure 2:**
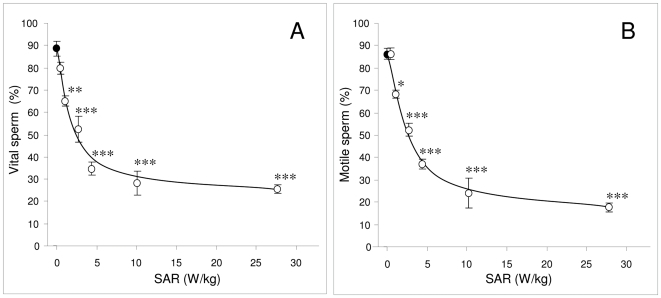
RF-EMR exposure reduces motility and vitality of human spermatozoa, in an SAR dependent manner. Percoll-purified spermatozoa (5×10^6^ cells) were suspended in 1 ml BWW in a 35 mm Petri dish and placed within the waveguide while control cells (closed circles) were placed outside the waveguide. Cells in the waveguide were exposed to 1.8 GHz RF-EMR at SAR levels of 0.4, 1.0 2.8 4.3 10.1 and 27.5 W/kg (open circles) for 16 h at 21°C. Both vitality and motility were reduced in a dose dependent manner. A, Vitality was significantly reduced at a SAR of 1.0 W/kg from 89%±3% to 65%±1% (**p<0.01). B, Motility was also significantly reduced at a SAR of 1.0 W/kg from 86%±2% to 68%±2% (*p<0.05). All results are based on 4 independent samples.

High quality spermatozoa selected in discontinuous Percoll gradients displayed a decline in both vitality and motility after exposure to RF-EMR in a dose- dependent manner. The control populations maintained an average vitality of 89%; however, significant reductions in vitality were observed at exposure levels as low as 1.0 W/kg (p<0.01) (Figure 2A). Similarly, the control populations maintained motilities at an average of 86% over the incubation period, however after exposure to RF-EMR at levels of 1.0 W/kg, motility was observed to significantly decrease to 68% (p<0.05) and decreased still further at higher SAR exposures (Figure 2B).

### Reactive Oxygen Species are central to the RF-EMR response

**Figure 3:**
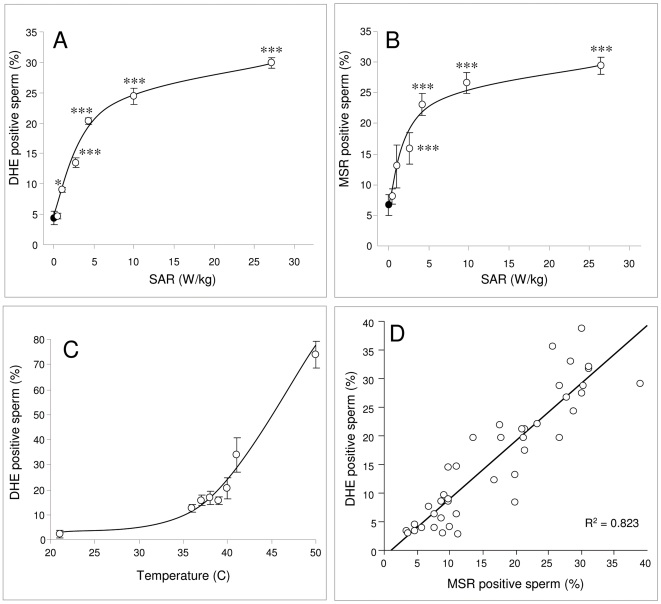
RF-EMR induces ROS generation in human spermatozoa, in an SAR-dependent manner unrelated to thermal effects. Percoll-purified spermatozoa (5×10^6^ cells) were suspended in 1 ml BWW in a 35 mm Petri dish and placed within the waveguide while control cells placed outside the waveguide (closed circles). Cells in the waveguide were exposed to 1.8 GHz RF-EMR at SAR levels between 0.4 and 27.5 W/kg (open circles) for 16 h at 21°C. Also, purified sperm cells were subjected to incubation temperatures ranging from 21°C–50°C for 2 h. As the power levels were increased, the cellular generation of ROS increased in a dose-dependent manner. ROS levels were also observed to increase as a result of incubation temperature, but such results were not significant until the temperature exceeded 40°C. A, ROS generation (DHE response) was significantly increased from control levels after exposure to 1.0 W/kg (*p<0.05) and above (***p<0.001). B, RF-EMR induces ROS generation by the sperm mitochondria as monitored by MSR; significant increases were observed at SAR values of 2.8 W/kg (***p<0.001) and above. All results are based on 4 independent samples. C, In order to control for thermal effects, the impact of temperature of cellular ROS generation was monitored; a significant increase in ROS generation was observed as temperatures rose above 40°C (p<0.001). D, Across the entire data set, the total level of ROS generation by human spermatozoa (DHE positive cells) was highly correlated with the level of ROS generation by the mitochondria (MSR positive cells: R^2^ = 0.823).

Exposure of human spermatozoa to RF-EMR over a range of SAR levels resulted in a dose-dependent activation of ROS generation, as detected by the DHE probe (Figure 3A). In this analysis, a significant increase in ROS positive cells was observed after exposure at 1.0 W/kg (p<0.05); thereafter ROS production rose rapidly with SAR values up to 4.3 W/kg and then began to plateau reaching a peak of 30% at the highest exposure levels assessed (Figure 3A). To determine whether such increases in ROS production might originate from the sperm mitochondria, MSR was employed as a probe. Spermatozoa exposed to increasing levels of RF-EMR, generated a significant, dose-dependent increase in ROS generation by the mitochondria. The response rose rapidly following RF-EMR exposure reaching statistical significance (p<0.001) at an SAR value 2.8 W/kg at which point 16% of the exposed cells were MSR positive. At SAR values above 4.3 W/kg, RF-EMR induced mitochondrial ROS begun to plateau reaching 30% at the maximal SAR values assessed (Figure 3B). By plotting the DHE positive cells against the MSR response for the entire data set (Figure 3D) we observed an extremely strong correlation (R^2^ = 0.823) between these signals, suggesting that a majority of the ROS production elicited by RF-EMR involved electron leakage from the mitochondrial electron transport chain.

In order to control for bulk thermal effects of RF-EMR exposure, spermatozoa were also incubated at temperatures ranging from 21°C–50°C for 2 h (Figure 3C). This analysis did reveal an effect of heat on free radical generation by human spermatozoa possibly due to the activation of an apoptotic response, however these effects were only significant above 40°C. Thus at the temperature at which these experiments were performed (21°C) the highest observed RF-EMR-induced temperature rise (+0.4°C at 27.5 W/kg), could not of itself account for the increased ROS response observed across the range of SAR settings evaluated in this study.

### RF-EMR induces oxidative DNA damage (8-OH-dG)

**Figure 4:**
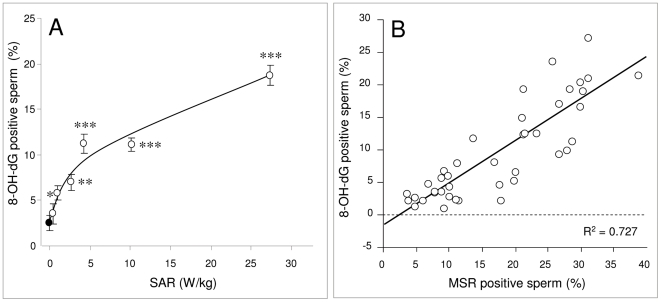
RF-EMR induces oxidative DNA damage in human spermatozoa. Following Percoll fractionation, 5×10^6^ high density, spermatozoa were suspended in 1 ml BWW. The cells were placed in 35 mm Petri dishes and placed inside a waveguide. 5×10^6^ cells in 1 ml BWW were placed outside the waveguide as a control (closed circle). The cells in the waveguide were exposed to 1.8 GHz RF-EMR at SAR levels between 0.4 and 27.5 W/kg (open circles) and all samples were incubated for 16 h at 21°C. Following incubation, Fe^2+^ and H_2_O_2_ was added to cells to act as a positive control, incubated for 1 h, then 100 µl 2 mM DTT/BWW solution was added and incubated for 45 min at 37°C. Cells were fixed and labeled with 100 µl charcoal purified anti-8-OH-dG, FITC tagged antibody at a dilution of 1∶50, incubated at 21°C for 1 h, washed and then assessed by flow cytometry. A, As the power levels were increased, the amount of oxidative DNA damage expressed also increased. A significant amount of oxidative DNA damage was observed in cells exposed to 2.8 W/kg (*p<0.05) RF-EMR and above (**p<0.01; ***p<0.001). Results are based on 4 independent samples. B, The levels of 8-OH-dG expression were positively correlated with the levels of ROS generation by the mitochondria (R^2^ = 0.727).

In order to determine whether the ROS generation induced on exposure of human spermatozoa to RF-EMR resulted in a state of oxidative stress, we monitored the expression of 8-hydroxy-2′-deoxyguanosine (8-OH-dG), a marker for oxidative damage to sperm DNA. As the SAR level was increased, the amount of oxidative DNA damage expressed in the spermatozoa became elevated (Figure 4A). A significant increase in 8-OH-dG expression became apparent at low SAR values (<5.0 W/kg) rising to a maximum of around 20% at the highest levels of exposure (27.5 W/kg). By plotting the 8-OH-dG positive cells against the MSR signal (Figure 4B) it was apparent that a strong positive correlation existed between the two parameters (R^2^ = 0.727); the higher the level of mitochondrial ROS generation, the greater the degree of oxidative DNA damage in the spermatozoa.

### RF-EMR induces DNA fragmentation in human spermatozoa

**Figure 5:**
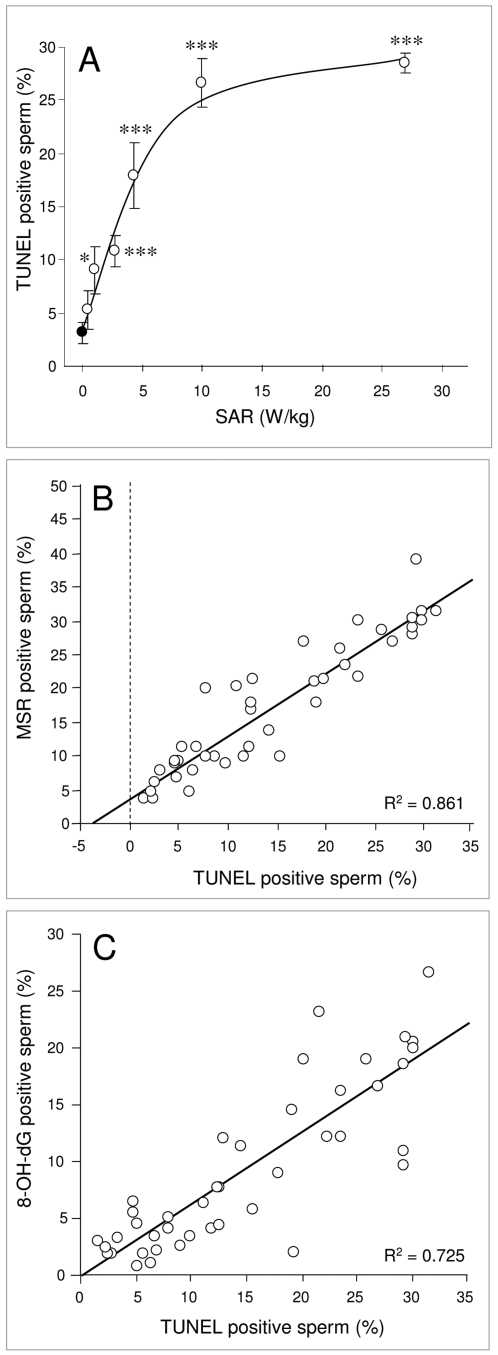
RF-EMR induces DNA fragmentation in human spermatozoa. Following Percoll fractionation, 5×10^6^ high density spermatozoa were resuspended in 1 ml BWW, pipetted into 35 mm Petri dishes and placed inside a waveguide. 5×10^6^ cells in 1 ml BWW were placed outside the waveguide as a control (closed circle). The cells in the waveguide were exposed to 1.8 GHz RF-EMR at SAR levels between 0.4 and 27.5 W/kg (open circles) and all samples were incubated for 16 h at 21°C. Following incubation, cells were fixed; DNase-I was used as a positive control. After 1 h incubation at 37°C, 50 µl of label and enzyme master mixes were added to the cells and incubated for 1 h at 37°C. Cells were then washed and assessed by flow cytometry. A, Significant levels of DNA fragmentation was observed in exposed spermatozoa at 2.8 W/kg (*p<0.05) and above (***p<0.001). B, DNA fragmentation was positively correlated with ROS production by the mitochondria as monitored by MSR (R^2^ = 0.861). C, 8-OH-dG was also positively correlated with DNA fragmentation (R^2^ = 0.725). Results are based on 4 independent samples.

To determine whether the oxidative DNA base damage precipitated by RF-EMR-induced ROS generation had any impact on DNA stand breaks in human spermatozoa, the terminal deoxynucleotidyl transferase dUTP nick end labeling (TUNEL) assay was utilized. As illustrated in Figure 5A, human spermatozoa responded to RF-EMR exposure, with a significant increase in DNA strand breaks at an SAR of 2.8 W/kg (p<0.05) that increased rapidly with rising SAR values and then reached a plateau so that at the highest SAR level assessed (27.5 W/kg), 29% of the cells expressed significant DNA fragmentation. This DNA damage was highly correlated with free radical generation by the sperm mitochondria giving a correlation coefficient of R^2^ = 0.861 (Figure 5B). Moreover, the level of DNA fragmentation was highly correlated with 8-OH-dG formation (R^2^ = 0.725; Figure 5C) such that sperm cells exhibiting high levels of oxidative DNA damage, also possessed high levels of DNA fragmentation.

## Discussion

While a high proportion of the male population suffers from infertility associated with defective sperm function [17], the etiology of this condition remains largely unresolved. Notwithstanding the general paucity of information in this area, recent studies have highlighted the interesting finding that male infertility patients are frequently characterized by high levels of DNA damage to their spermatozoa [18]. In light of these data, we have hypothesized that the disruption of sperm fertilizing potential and the concomitant presence of high levels of DNA damage in the sperm nucleus involves a common causative mechanism in the form of oxidative stress [19].

Oxidative stress has been known for some time to limit the fertilizing potential of human spermatozoa through the induction of peroxidative damage to the sperm plasma membrane [13], [20]. Oxidative stress is also known to be associated with DNA damage in human spermatozoa [21]. Furthermore, the source of the free radicals responsible for generating such stress appears to be the mitochondria [15]. However, the factors responsible for inducing the mitochondria to leak electrons and propagate the production of ROS have not been elucidated. The research described in this article suggests that one of the key environmental factors involved in the stimulation of sperm mitochondria to produce high levels of ROS, might be excess exposure to RF-EMR from sources such as mobile phones.

In a pilot study, human spermatozoa were found to respond to RF-EMR (at 1.8 GHz with a SAR of 27.5 W/kg) with a range of negative changes including dramatic declines in both sperm vitality and motility. We also observed significant increases in both cytoplasmic ROS levels (DHE) as well as mitochondrial ROS levels (MSR) after RF-EMR exposure. We have previously shown that the chemical induction of mitochondrial ROS production with rotenone can precipitate a state of oxidative stress leading to high levels of lipid peroxidation and a loss of sperm motility [15]. Therefore, these data highlight the particular vulnerability of human spermatozoa to oxidative attack and the potential significance of sperm mitochondria in the generation of free radicals.

To assess whether similar effects could be observed at lower power densities, closer to the SAR values associated with mobile phones (0.5–1.5 W/kg) a dose-dependent analysis was conducted. In addition to the conventional assessments of motility and vitality, assays were included to assess the potential for RF-EMR to induce sperm DNA damage and further, whether the DNA damage was oxidative in nature. Confirmation of the detrimental effects of RF-EMR on human sperm was again observed. Over the power density range employed, a significant (*P*<0.001) dose-dependent response for all sperm parameters was observed, including motility, vitality, ROS generation by the whole cell, ROS generation by the mitochondria, oxidative DNA damage and DNA fragmentation. Furthermore, the profiles of all the observed effects with respect to SAR were intriguingly similar, suggesting a common underlying mechanism.

Specifically, all of the responses examined showed an extremely rapid change at low SAR exposures that then reached a plateau at a point where around 30% of the sperm population was affected. This suggests that while we were careful to use only Percoll-purified, high quality spermatozoa in this analysis, there exists within this cell population, a cohort of spermatozoa that are particularly vulnerable to the induction of oxidative stress by RF-EMR. These spermatozoa may have compromised mitochondria, poorly remodeled chromatin or a combination of such factors [15], [22]. Heterogeneity within the sperm population is a feature of the human condition. However, this does not mean that a majority of spermatozoa would not, ultimately, be affected by RF-EMR in vivo; much would depend on the duration of exposure. In vitro, we are limited by the inability of human spermatozoa to survive for more than 24 hours in a simple defined culture medium. In vivo, spermatozoa may take up to a week to move from the seminiferous tubules in the testes to the cauda epididymis and during the whole of this time they would be vulnerable to RF-EMR exposure [23].

We recognize that these studies were conducted using spermatozoa suspended in a simple defined culture medium rather than the epididymal plasma in which they would be suspended in vivo. Nevertheless the fact that effects on sperm quality have previously been observed in both whole animal radiation experiments [3] and in epidemiological studies of human subjects exposed to various levels of mobile phone radiation [5], [7], [24], emphasizes the biological and clinical relevance of these findings. Moreover, another recent study has found that exposing human spermatozoa to mobile phone radiation for 1 hour leads to significant declines in motility and vitality in concert with an increase in cellular reactive oxygen species generation [25]. The levels of RFEMR exposure were not quantified in this study nor were the sources of ROS identified. Nevertheless, these findings reinforce the general conclusions generated in this paper, particularly with respect to central role played by oxidative stress. The ever-increasing prevalence of mobile communications technology means that humans are now exposed to higher amounts of RF-EMR than ever before. Mobile phones are commonly carried in bags or in pockets in very close proximity to the body. In addition to this, these devices can be stored adjacent to the same part of the body for extended periods of time. In this context, exposure of the male reproductive system to RF-EMR is clearly a significant issue.

The particular significance of the present study is that it not only demonstrates a direct effect of RF-EMR on sperm motility, vitality and DNA integrity but also identifies a potential causative mechanism involving electron leakage from the mitochondrial electron transport chain and the induction of oxidative DNA damage. In part, these mechanistic insights have been achieved because the cell type used in these studies, the human spermatozoon, has an extremely simple cellular architecture, lacking significant cytosol and possessing few cellular organelles other than the sperm nucleus, flagellum and mitochondria. One consequence of this structure is that these cells are uniquely vulnerable to oxidative stress. Moreover, such stress is already known to induce the functional and structural lesions observed in this study including both a loss of motility mediated by peroxidative damage to the sperm plasma membrane, as well as the formation of DNA base adducts in the sperm nucleus that ultimately lead to DNA fragmentation [26], [27].

Notwithstanding the specialized nature of mammalian spermatozoa, the mechanisms suggested by this study may also apply to RF-EMR-mediated damage in other cell types. The RF-EMR used for communications, including mobile phone networks, is not of high enough power to be classed as ionizing radiation. The latter has sufficient energy to pull away electrons, dramatically altering the properties of affected molecules and typically creating extremely reactive radical species. RF-EMR does not contain sufficient energy for these processes. Nevertheless, this form of radiation may have other effects on larger scale systems such as cells and organelles, which stem from the perturbation of charged molecules and the disruption of electron flow [28], [29]. Mitochondria have one of the largest standing membrane potentials in the body and their energetic functions are entirely dependent on the regulated movement of electrons and protons within the inner mitochondrion membrane. Theoretically, such fluxes might be susceptible to disruptions in local electric fields induced by RF-EMR, offering a potential link between this form of radiation and the non-thermal biological effects observed in this study.

This study clearly demonstrates that RF-EMR can damage sperm function via mechanisms that involve the leakage of electrons from the mitochondria and the creation of oxidative stress. These findings have immediate implications for the high rates of male infertility seen in our species, a majority of which is idiopathic. Furthermore, the fact that sperm DNA is damaged by this form of radiation has additional implications for the health and wellbeing of children born to fathers who have experienced high levels of occupational or environmental exposure to RF-EMR around the time of conception. Overall, these finding raise a number of related health policy and patient management issues that deserve our immediate attention. Specifically we recommend that men of reproductive age who engage in high levels of mobile phone use, do not keep their phones in receiving mode below waist level.

## Methods

### Ethics Statement

This study was conducted according to the principles expressed in the Declaration of Helsinki. The study was approved by the University of Newcastle (H-712-0799). All patients provided written informed consent for the collection of samples and subsequent analysis.

### Reagents and Solutions

All chemicals and reagents used in this research were obtained from Sigma Aldrich (Sigma Chemical Co., St. Louis, MO) unless stated otherwise. All reagents used were of research grade. All fluorescent probes were purchased from Molecular Probes Inc. (Eugene, OR). Biggers, Whitten and Whittingham (BWW) media supplemented with 1 mg/ml polyvinyl alcohol (PVA) was used in all experiments [30]. It was prepared fresh as required and kept at 37°C with an osmolarity in the range of 290–310 mOsm/kg.

### Human spermatozoa

Institutional and State Government ethical approval was secured for the use of human semen samples for this research. The donors were students from the University of Newcastle donor program who had no known prior male reproductive pathologies including varicocele and infection. From this pool, 22 normozoospermic donors were used in this study. The average (±SEM) age of these donors was 24.1±1.1 y. After allowing at least 30 min for liquefaction to occur, spermatozoa were separated from seminal plasma on a discontinuous two-step Percoll gradient, as described [16]. The isolated spermatozoa were washed with 10 ml BWW, centrifuged at 600× g for 15 min and finally resuspended in HEPES-buffered BWW at a concentration of 20× 10^6^/ml supplemented with 1 mg/ml PVA. After acquiring each sperm fraction, the vitality, motility and cell density of the spermatozoa were evaluated. Vitality was determined by transferring 5 µl of each cell fraction onto a microscope slide followed by the addition of 5 µl of 0.5% eosin; the percentage of non-viable cells staining pink was then assessed by light microscopy. Motility was assessed by transferring 6 µl of the same sample onto a slide which was then covered with a coverslip and examined by phase contrast microscopy. For both the vitality and motility assessments, 100 cells were counted and the results expressed as a percentage.

### Radio Frequency Electromagnetic Radiation and Waveguide

In this study, a cylindrical waveguide copied from the design by Gajda *et al* [31] was constructed such that 1.8 GHz radiation could propagate along the waveguide and also so that 35 mm Petri dishes could be accommodated within the waveguide. To produce the radiation, a 3 GHz function generator (E4431B; Agilent, Palo Alto, CA) was used to generate a pure tone of 1.8 GHz. This signal was amplified by a linear radio-frequency (RF) amplifier and the amplifier output was split and connected through a matching network to antennae in the waveguide. The antenna matching circuit was tuned for maximum energy transfer to the antenna. The waveguide was encased in a brass mesh Faraday cage and the end was filled with 15 cm thick carbon-impregnated foam (RFI Industries, Bayswater, Victoria, Australia), which absorbs RF radiation, minimizing the reflection of radiation back into the waveguide and reducing the RF power by more than 50 dB outside the Faraday cage compared to the power at the amplifier output. A spectrum analyser (Advantest, Tokyo, Japan) connected to a Hameg HZ530 E-field probe (Hameg GmbH, Mainhausen, Germany) was used to check radiation levels and frequency prior to irradiation. The SAR values for the irradiations were calibrated by measuring the temperature rise in saline solution at power levels 20 dB or 100× higher than for the normal irradiations. The calibration procedure is complicated because (i) the saline solution loses heat energy to the surroundings at the same time as it is heated by the RF radiation and (ii) the temperature rise must be measured by an electronic thermometer to achieve the 0.1°C resolution required; however, the RF field interfered with the thermometer operation. As a consequence of these factors, the saline temperature was measured as a function of the time delay after the RF field was turned off and the temperature change extrapolated back to zero delay. Multiple measurements were made for RF irradiation times varying from 15 to 120 s and temperature increases up to 2.2°C above the ambient temperature were measured. After allowing for heat losses to the surroundings, the power level of 38.8 dBm at the amplifier output used in these measurements gave rise to a saline temperature rise of 0.053±0.008°Cs^−1^, giving a SAR of 220±33 Wkg^−1^. This error is similar to the variation in SAR observed in reference paper as a function of probe position [31]. The values of SAR reported in this paper were calculated from the above SAR, linearly scaled by the amplifier output power.

Following sperm purification and initial analysis, the high density Percoll fraction was prepared as a 1 ml suspension in BWW containing 5×10^6^ cells and transferred into 35 mm Petri dishes. The cells to be irradiated were placed inside the waveguide while the control cells where placed adjacent to the waveguide but outside the Faraday cage. The SAR levels (0.4–27.5 W/kg) were fixed by setting the RF source to the appropriate dBm value. For all RF-EMR exposures (and respective controls) spermatozoa were incubated at room temperature (21°C) for a period of 16 h. Motility and vitality was measured prior to as well as after treatment. ROS and DNA damage assays were completed on both the exposed cells and respective controls after incubation.

### Dihydroethidium Assay

Dihydroethidium (DHE) is a poorly fluorescent 2-electron reduction product of ethidium that on oxidation produces DNA sensitive fluorochromes that generate a red nuclear fluorescence when excited at 510 nm. The results obtained with this probe have been validated as a measure of the ability of human spermatozoa to generate ROS, including definitive identification of the superoxide anion [32]. For the assay, DHE and the vitality stain, SYTOX^®^ Green (Molecular Probes), were diluted in BWW/PVA and added to 2×10^6^ spermatozoa in a final volume of 200 µl comprising 175 µl of purified sperm suspension, 5 µl of test compound and 20 µl of the DHE:SYTOX^®^ green mixture to give final concentrations of 2 µM DHE and 0.5 µM SYTOX^®^ green. The cells were then incubated in the dark at 37°C for 15 min, washed once (600×*g* for 5 min) and the resultant red and green fluorescence measured on a FACSCalibur flow cytometer (Becton Dickinson, San Jose, CA), as described [32]. The unstained control displayed 0.09%±0.03% DHE positivity, the DHE positive control (treated with 100 µM arachidonic acid) displayed 99%±1% DHE positivity and the SyG positive control (frozen-thawed cells) displayed 98%±1% SyG positivity. The inclusion of SyG in this assay ensured that the production of ROS was only being assessed in live cells.

### MitoSOX Red (MSR) Assay

MSR is a poorly fluorescent compound similar to DHE but carrying a charge that results in the selective accumulation of this probe within the mitochondria. Following reaction with the superoxide anion, MSR produces DNA sensitive fluorochromes that generate a red fluorescence when excited at 510 nm that can be detected by flow cytometry.

As with the DHE assay, SyG was used in order to ensure that only live cells were evaluated in this assay. MSR and SyG stock solutions (in DMSO) were diluted in BWW/PVA and 20 µl of each added to each treatment to give final concentrations of 2 µM and 0.05 µM respectively in a final volume of 200 µl. The cells were incubated at 37°C away from light for 15 min, centrifuged at 600× *g* for 5 min and the supernatant discarded. The pellet was then washed in 200 µl BWW/PVA, resuspended in 1 ml of this medium and transferred to 5 ml FACS tubes for analysis by flow cytometry. [15] The unstained control displayed 0.66%±0.32% MSR positivity, the MSR positive control (treated with 100 µM arachidonic acid) displayed 96%±3% MSR positivity and the SyG control displayed 96%±1% SyG positivity.

### Assay for 8-hydroxy-2′-deoxyguanosine (8-OH-dG)

The formation of the 8-OH-dG base lesion, which is a biomarker for oxidative stress, was measured using an anti-8-OH-dG antibody (supplied in the Biotrin OxyDNA test Kit, Biotrin International Ltd, Dublin, Ireland) which was conjugated with a fluorescent label, fluorescein isothiocyanate (FITC). The level of FITC fluorescence was then measured using flow cytometry. For the positive control, spermatozoa were incubated for 1 h at room temperature with H_2_O_2_ (2 mM) and FeCl_2_•4H_2_O (1 mM) in a final volume of 200 µl BWW. The initial H_2_O_2_ concentration was determined by measuring absorbance at 240 nm (ε = 43.6 M^−1^ cm^−1^). The cells were then washed twice in BWW, resuspended in 100 µl of 2 mM dithiothreitol (DTT) in BWW and incubated for 45 min at 37°C. After centrifugation at 600× *g* for 5 min, the cells were then fixed by resuspending the pellet in 100 µl Phosphate Buffered Saline (PBS) and 100 µl 4% paraformaldehyde and incubated at 4°C for 15 min. The cells were then washed in PBS and stored in 200 µl 0.1 M glycine at 4°C and stored for a maximum of 1 week. Fixed cells were washed and resuspended in 100 µl 0.2% Triton-X and incubated at room temperature for 15 min. Cells were then washed in Wash Solution (Biotrin OxyDNA test Kit, Biotrin International Ltd.) and 50 µl blocking solution (Biotrin OxyDNA test Kit, Biotrin International Ltd.) added before incubation at 37°C for 1 h. The anti-8-OH-dG antibody was further purified by adding approximately 1 mg of activated charcoal powder, followed by incubation at room temperature for 1 h and centrifugation at 600× *g* for 5 min. This step was repeated once more for complete removal of the charcoal. The supernatant containing the purified antibody was then added in a 1∶50 dilution to the fixed cells in wash solution with a final volume of 100 µl. Finally, cells were washed twice, resuspended in 1 ml PBS and transferred to 5 ml FACS tubes for flow cytometric analysis. The unstained control and positive (H_2_O_2_/Fe^2+^) control displayed 0.09%±0.02% and 97%±1% 8-OH-dG positivity, respectively.

### TUNEL Assay

Spermatozoa were centrifuged (600× *g* for 4 min) before resuspending the pellet in 100 µl of fresh permeabilization solution (10 mg sodium citrate, 10 µl triton-X in 10 ml dH_2_O) and incubating for 2 min at 4°C. The cells were then centrifuged (600× *g* for 4 min) and the pellets washed with PBS. The positive control samples were treated with 100 µl of DNase I (1 mg/ml) for 30 min at 37°C in a humid environment. TUNEL labeling was achieved with the In Situ Cell Death Detection Kit (Roche Diagnostics, Indianapolis, IN) according to the manufacturer's instructions. Cells were then washed twice in PBS, diluted to a final volume of 500 µl in PBS and kept in the dark for analysis using flow cytometry.

### Analysis by Flow Cytometry

For flow cytometry analysis, Falcon 35 (2008) 5 mL polystyrene round bottom tubes were used for aspirating the sample into the fluorescence activated cell sorter (FACS). At least 5,000 cells were analyzed for each assay using a FACS™ calibur (Becton Dickinson) and the gates were set, based on forward and side scatter, such that only spermatozoa were assessed [15]. Fluorescence was measured upon excitation by a 15 mW argon-ion laser at 488 nm and was paired with emission measurements using 530/30 band pass (green/FL-1), 585/42 band pass (red/FL-2) and >670 long pass (far red/FL-3) filters. The FL-1 and the FL-2 filters were used for the vitality stain (SyG) and ROS stain (DHE) respectively. For TUNEL and 8-OH-dG analysis, only the FL-1 filter was used and for these assays. The software used to analyze the data was CellQuest Pro (BD Biosciences, San Jose, CA).

### Statistics

All experiments were repeated at least 3 times on independent samples and the results analyzed by ANOVA using the SuperANOVA programme (Abacus Concepts Inc, CA) on a MacIntosh G5 computer; post hoc comparison of group means was determined by Fisher's PLSD test. Differences with a *P* value of <0.05 were regarded as significant. All data are presented as the mean value±SEM.
