# Correction: Mobile Phone Radiation Induces Reactive Oxygen Species Production and DNA Damage in Human Spermatozoa *In Vitro*

**DOI:** 10.1371/annotation/9a8a0172-3850-4059-b852-72c330769c1b

**Published:** 2013-03-11

**Authors:** Geoffry N. De Iuliis, Rhiannon J. Newey, Bruce V. King, R. John Aitken

After a re-evaluation of the original data for Figure 1 in this article, it has come to our attention that the data set for the panel 1D was incorrectly transcribed from its source, as a result, we are providing a corrected file for Figure 1, the correct values reflecting the changes made in panel D are: 28% ± 1% for the exposed cells and 5% ± 1% for the control cells, with a p value of 0.0056.

Figure 1: 

**Figure:**
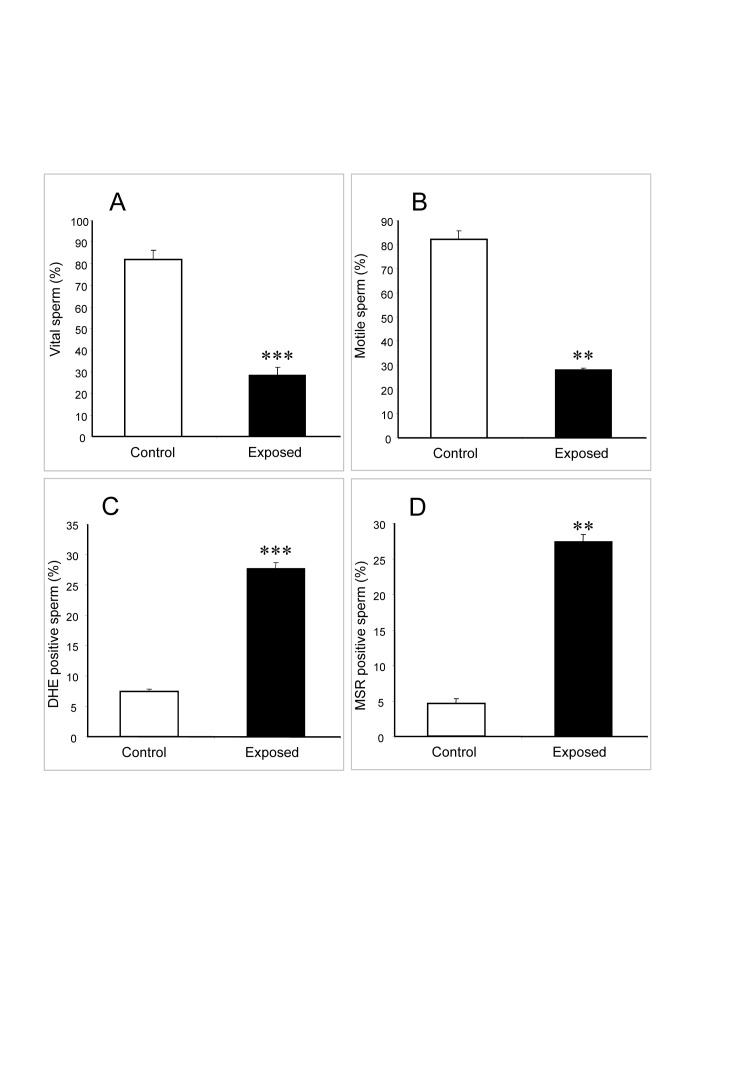


In addition, the number of biological replicates reported in the figure legends is incorrect, the data are based on 3 replicates and not 4 as stated in the article. In the figure legends for figures 1, 2, 3, 4 and 5, the number of independent samples should thus be changed from 4 to 3. 

